# 2,3-Diamino­pyridinium 3-carb­oxy-4-hy­droxy­benzene­sulfonate monohydrate

**DOI:** 10.1107/S160053681104445X

**Published:** 2011-10-29

**Authors:** Madhukar Hemamalini, Jia Hao Goh, Hoong-Kun Fun

**Affiliations:** a X-ray Crystallography Unit, School of Physics, Universiti Sains Malaysia, 11800 USM, Penang, Malaysia

## Abstract

In the title hydrated mol­ecular salt, C_5_H_8_N_3_^+^·C_7_H_5_O_6_S^−^·H_2_O, the ion pairs and water mol­ecules are connected by N—H⋯O, O—H⋯O and C—H⋯O hydrogen bonds, thereby forming a three-dimensional network. There is an intra­molecular O—H⋯O hydrogen bond in the 3-carb­oxy-4-hy­droxy­benzene­sulfonate anion, which generates an *S*(6) ring motif.

## Related literature

For background to 5-sulfosalicylic acid and related compounds, see: Marzotto *et al.* (2001 ▶); Onoda *et al.* (2001 ▶); Baskar Raj *et al.* (2003 ▶). For hydrogen-bond motifs, see: Bernstein *et al.* (1995 ▶). For bond-length data, see: Allen *et al.* (1987 ▶). For the stability of the temperature controller used in the data collection, see: Cosier & Glazer (1986 ▶). 
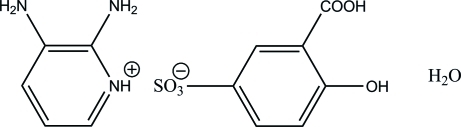


## Experimental

### 

#### Crystal data

C_5_H_8_N_3_^+^·C_7_H_5_O_6_S^−^·H_2_O
*M* *_r_* = 345.33
Monoclinic, 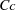

*a* = 7.0407 (7) Å
*b* = 15.5775 (16) Å
*c* = 13.6244 (12) Å
β = 101.491 (2)°
*V* = 1464.3 (2) Å^3^
*Z* = 4
Mo *K*α radiation
μ = 0.26 mm^−1^
*T* = 100 K
0.36 × 0.31 × 0.08 mm

#### Data collection

Bruker APEXII DUO CCD diffractometer
Absorption correction: multi-scan (*SADABS*; Bruker, 2009 ▶) *T*_min_ = 0.910, *T*_max_ = 0.981
6890 measured reflections
3880 independent reflections
3645 reflections with *I* > 2σ(*I*)
*R*_int_ = 0.025

#### Refinement

*R*[*F* ^2^ > 2σ(*F* ^2^)] = 0.033
*wR*(*F* ^2^) = 0.087
*S* = 1.03
3880 reflections
268 parameters
2 restraints
All H-atom parameters refined
Δρ_max_ = 0.33 e Å^−3^
Δρ_min_ = −0.19 e Å^−3^
Absolute structure: Flack (1983 ▶), 1770 Friedel pairs
Flack parameter: −0.02 (5)

### 

Data collection: *APEX2* (Bruker, 2009 ▶); cell refinement: *SAINT* (Bruker, 2009 ▶); data reduction: *SAINT*; program(s) used to solve structure: *SHELXTL* (Sheldrick, 2008 ▶); program(s) used to refine structure: *SHELXTL*; molecular graphics: *SHELXTL*; software used to prepare material for publication: *SHELXTL* and *PLATON* (Spek, 2009 ▶).

## Supplementary Material

Crystal structure: contains datablock(s) global, I. DOI: 10.1107/S160053681104445X/hb6457sup1.cif

Structure factors: contains datablock(s) I. DOI: 10.1107/S160053681104445X/hb6457Isup2.hkl

Supplementary material file. DOI: 10.1107/S160053681104445X/hb6457Isup3.cml

Additional supplementary materials:  crystallographic information; 3D view; checkCIF report

## Figures and Tables

**Table 1:** Hydrogen-bond geometry (Å, °)

*D*—H⋯*A*	*D*—H	H⋯*A*	*D*⋯*A*	*D*—H⋯*A*
N1—H1*N*1⋯O1*W*^i^	0.91 (3)	1.95 (3)	2.806 (3)	158 (3)
N2—H1*N*2⋯O1^i^	0.90 (3)	2.48 (3)	3.222 (2)	140 (2)
N2—H1*N*2⋯O1*W*^i^	0.90 (3)	2.28 (3)	3.060 (3)	145 (2)
N2—H2*N*2⋯O2^ii^	0.86 (3)	2.10 (3)	2.963 (2)	177 (3)
N3—H1*N*3⋯O2^ii^	0.89 (3)	2.10 (3)	2.970 (3)	168 (3)
N3—H2*N*3⋯O4^iii^	0.92 (3)	2.08 (3)	2.980 (3)	167 (3)
O1—H1*O*1⋯O6	1.03 (3)	1.75 (3)	2.625 (2)	141 (2)
O5—H1*O*5⋯O3^iv^	0.84 (3)	1.87 (3)	2.655 (2)	155 (3)
O1*W*—H1*W*1⋯O3^v^	0.89 (3)	1.94 (3)	2.757 (3)	151 (2)
O1*W*—H2*W*1⋯O4^iii^	0.98 (6)	1.89 (6)	2.838 (3)	162 (4)
C7—H7⋯O3^vi^	0.95 (3)	2.56 (3)	3.477 (2)	163 (2)

Symmetry codes: (i) 
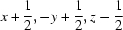
; (ii) 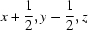; (iii) 
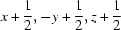
; (iv) 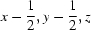; (v) 
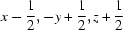
; (vi) 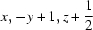.

**Fig. 1.**
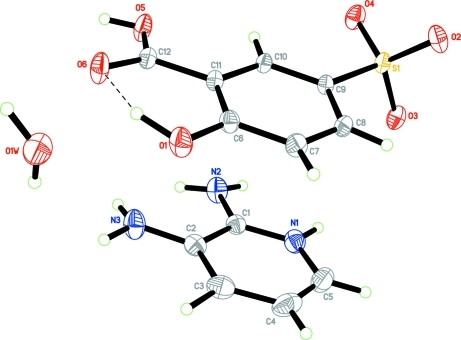
The asymmetric unit of the title compound, showing 50% probability displacement ellipsoids. Intramolecular hydrogen bonds shown by dashed lines.

**Fig. 2.**
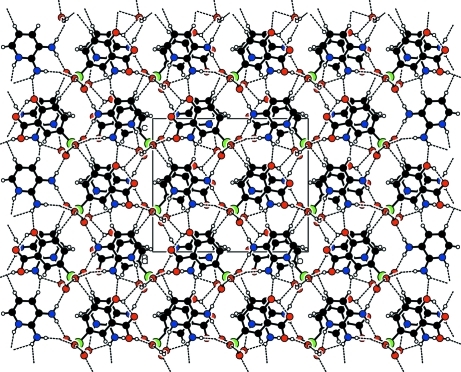
The crystal packing of title compound (I).

## supplementary crystallographic information

### Comment

5-sulfosalicylic acid, (5-SSA), has been known for a long time to possess anti-inflammatory activity. When it forms complexes with metals, its biological activity is greatly enhanced (Marzotto *et al.*, 2001).  Hydrogen-bonding patterns involving sulfonate groups in biological systems and metal complexes are of current interest (Onoda *et al.*, 2001). Such interactions can be utilized for designing supramolecular architectures (Baskar Raj *et al.*, 2003).  With the aim of gaining more insight into hydrogen-bonding interactions involving 5-SSA and pyridine derivatives, we report here the molecular and supramolecular structure of the title compound.

The asymmetric unit of (I) contains a 2,3-diaminopyridinium cation, a sulfosalicylate anion and a water molecule (Fig. 1). The 2,3-di aminopyridinium cation is planar, with a maximum deviation of 0.015 (2) Å for atom C1. The dihedral angle between the pyridine (N1/C–C5) and the benzene (C6–C11) ring is 6.09 (9)°. The protonated N1 atom has lead to a slight increase in the C1—N1—C5 angle to 124. 4(2)°.  The bond lengths (Allen *et al.*, 1987) and angles are within normal ranges.  There is an intramolecular O—H···O hydrogen bond in the 3-carboxy-4-hydroxy benzenesulfonate anion, which generates an *S*(6) (Bernstein *et al.*, 1995) ring motif.

In the crystal (Fig. 2), the ion-pairs and water molecules are connected *via* N—H···O, O—H···O and C—H···O hydrogen bonds (Table 1), forming a three-dimensional network.

### Experimental

Hot methanol solutions (20 ml) of 2,3-diaminopyridine (52 mg, Aldrich) and 5-sulfosalicylic acid (54. 5 mg, Merck) were mixed and warmed over a heating magnetic stirrer for 5 minutes. The resulting solution was allowed to cool slowly at room temperature.  Brown plates of the title compound appeared from the mother liquor after a few days.

### Refinement

All hydrogen atoms were located from a difference Fourier maps and refined freely [N–H = 0.86 (3)–0.92 (3) Å; O–H = 0.90 (3)– 1.02 (3) Å and C–H = 0.91 (4)–1.06 (3) Å]. 1770 Friedel pairs were used to determine the absolute structure.

### Crystal data

**Table:**

C_5_H_8_N_3_^+^·C_7_H_5_O_6_S^−^·H_2_O	*F*(000) = 720
*M**_r_* = 345.33	*D*_x_ = 1.566 Mg m^−^^3^
Monoclinic, *C**c*	Mo *K*α radiation, λ = 0.71073 Å
Hall symbol: C -2yc	Cell parameters from 3903 reflections
*a* = 7.0407 (7) Å	θ = 3.0–32.4°
*b* = 15.5775 (16) Å	µ = 0.26 mm^−^^1^
*c* = 13.6244 (12) Å	*T* = 100 K
β = 101.491 (2)°	Plate, brown
*V* = 1464.3 (2) Å^3^	0.36 × 0.31 × 0.08 mm
*Z* = 4	

### Data collection

**Table:**

Bruker APEXII DUO CCD diffractometer	3880 independent reflections
Radiation source: fine-focus sealed tube	3645 reflections with *I* > 2σ(*I*)
graphite	*R*_int_ = 0.025
φ and ω scans	θ_max_ = 30.0°, θ_min_ = 3.0°
Absorption correction: multi-scan (*SADABS*; Bruker, 2009)	*h* = −9→9
*T*_min_ = 0.910, *T*_max_ = 0.981	*k* = −20→21
6890 measured reflections	*l* = −18→19

### Refinement

**Table:**

Refinement on *F*^2^	Secondary atom site location: difference Fourier map
Least-squares matrix: full	Hydrogen site location: inferred from neighbouring sites
*R*[*F*^2^ > 2σ(*F*^2^)] = 0.033	All H-atom parameters refined
*wR*(*F*^2^) = 0.087	*w* = 1/[σ^2^(*F*_o_^2^) + (0.0525*P*)^2^] where *P* = (*F*_o_^2^ + 2*F*_c_^2^)/3
*S* = 1.03	(Δ/σ)_max_ = 0.001
3880 reflections	Δρ_max_ = 0.33 e Å^−^^3^
268 parameters	Δρ_min_ = −0.19 e Å^−^^3^
2 restraints	Absolute structure: Flack (1983), 1770 Friedel pairs
Primary atom site location: structure-invariant direct methods	Flack parameter: −0.02 (5)

### Special details

**Table:**

Experimental. The crystal was placed in the cold stream of an Oxford Cryosystems Cobra open-flow nitrogen cryostat (Cosier & Glazer, 1986) operating at 100.0 (1) K.
Geometry. All s.u.'s (except the s.u. in the dihedral angle between two l.s. planes) are estimated using the full covariance matrix. The cell s.u.'s are taken into account individually in the estimation of s.u.'s in distances, angles and torsion angles; correlations between s.u.'s in cell parameters are only used when they are defined by crystal symmetry. An approximate (isotropic) treatment of cell s.u.'s is used for estimating s.u.'s involving l.s. planes.
Refinement. Refinement of F^2^ against ALL reflections. The weighted R-factor wR and goodness of fit S are based on F^2^, conventional R-factors R are based on F, with F set to zero for negative F^2^. The threshold expression of F^2^ > 2σ(F^2^) is used only for calculating R-factors(gt) etc. and is not relevant to the choice of reflections for refinement. R-factors based on F^2^ are statistically about twice as large as those based on F, and R- factors based on ALL data will be even larger.

### Fractional atomic coordinates and isotropic or equivalent isotropic displacement parameters (Å^2^)

**Table:**

	*x*	*y*	*z*	*U*_iso_*/*U*_eq_	
N1	0.9025 (3)	0.36254 (11)	−0.04106 (15)	0.0419 (4)	
N2	0.7986 (3)	0.24446 (13)	−0.14072 (14)	0.0438 (4)	
N3	0.7871 (3)	0.14977 (13)	0.03470 (16)	0.0482 (4)	
C1	0.8513 (2)	0.27950 (12)	−0.04911 (15)	0.0328 (3)	
C2	0.8521 (2)	0.23206 (13)	0.04046 (14)	0.0349 (4)	
C3	0.9159 (3)	0.27470 (17)	0.12994 (16)	0.0465 (4)	
C4	0.9699 (3)	0.36114 (19)	0.1331 (2)	0.0551 (6)	
C5	0.9615 (3)	0.40443 (15)	0.0472 (2)	0.0516 (5)	
S1	0.36330 (5)	0.48126 (2)	−0.18842 (3)	0.02841 (9)	
O1	0.4248 (2)	0.23706 (10)	0.14554 (10)	0.0430 (3)	
O2	0.2649 (2)	0.55517 (9)	−0.15649 (10)	0.0430 (3)	
O3	0.5610 (2)	0.50245 (9)	−0.19835 (11)	0.0408 (3)	
O4	0.2514 (2)	0.44146 (9)	−0.27754 (10)	0.0402 (3)	
O5	0.2302 (2)	0.15275 (9)	−0.14779 (11)	0.0444 (3)	
O6	0.2779 (3)	0.11944 (10)	0.01427 (12)	0.0484 (4)	
C6	0.4112 (2)	0.28932 (11)	0.06627 (12)	0.0305 (3)	
C7	0.4712 (3)	0.37427 (12)	0.08458 (13)	0.0337 (3)	
C8	0.4596 (2)	0.43146 (11)	0.00715 (13)	0.0312 (3)	
C9	0.3843 (2)	0.40512 (10)	−0.09102 (11)	0.0253 (3)	
C10	0.3256 (2)	0.32121 (10)	−0.11074 (12)	0.0258 (3)	
C11	0.3407 (2)	0.26183 (10)	−0.03244 (12)	0.0264 (3)	
C12	0.2809 (2)	0.17180 (11)	−0.05209 (13)	0.0306 (3)	
O1W	0.3732 (3)	0.08650 (11)	0.25895 (15)	0.0533 (4)	
H3	0.930 (4)	0.2373 (19)	0.1958 (19)	0.046 (7)*	
H4	1.007 (5)	0.389 (2)	0.193 (3)	0.069 (9)*	
H5	1.011 (5)	0.463 (2)	0.039 (2)	0.067 (9)*	
H7	0.515 (4)	0.3983 (18)	0.149 (2)	0.041 (6)*	
H8	0.510 (4)	0.4874 (19)	0.021 (2)	0.043 (7)*	
H10	0.271 (3)	0.3039 (15)	−0.1722 (17)	0.025 (5)*	
H1N1	0.899 (5)	0.393 (2)	−0.098 (2)	0.057 (8)*	
H1N2	0.820 (4)	0.277 (2)	−0.192 (2)	0.053 (8)*	
H2N2	0.793 (4)	0.1894 (19)	−0.146 (2)	0.042 (6)*	
H1N3	0.763 (4)	0.1187 (19)	−0.021 (2)	0.047 (7)*	
H2N3	0.794 (4)	0.1179 (18)	0.092 (2)	0.044 (6)*	
H1O1	0.354 (4)	0.181 (2)	0.122 (2)	0.053 (7)*	
H1O5	0.203 (5)	0.100 (2)	−0.149 (2)	0.057 (8)*	
H1W1	0.265 (5)	0.055 (2)	0.250 (2)	0.053 (8)*	
H2W1	0.490 (8)	0.071 (4)	0.234 (4)	0.110 (16)*	

### Atomic displacement parameters (Å^2^)

**Table:**

	*U*^11^	*U*^22^	*U*^33^	*U*^12^	*U*^13^	*U*^23^
N1	0.0392 (8)	0.0377 (9)	0.0515 (10)	0.0004 (6)	0.0154 (7)	0.0051 (7)
N2	0.0607 (10)	0.0399 (10)	0.0340 (8)	0.0010 (7)	0.0169 (7)	0.0017 (7)
N3	0.0721 (12)	0.0387 (9)	0.0373 (9)	−0.0017 (8)	0.0194 (8)	0.0054 (8)
C1	0.0297 (7)	0.0335 (8)	0.0366 (8)	0.0024 (6)	0.0103 (6)	0.0016 (7)
C2	0.0330 (8)	0.0385 (10)	0.0352 (9)	0.0044 (6)	0.0116 (7)	0.0007 (8)
C3	0.0452 (10)	0.0592 (13)	0.0343 (10)	0.0045 (9)	0.0058 (8)	0.0009 (9)
C4	0.0444 (11)	0.0639 (15)	0.0538 (14)	−0.0043 (9)	0.0017 (9)	−0.0239 (12)
C5	0.0415 (9)	0.0405 (11)	0.0723 (15)	−0.0054 (8)	0.0105 (10)	−0.0113 (10)
S1	0.03858 (18)	0.02089 (16)	0.02544 (16)	−0.00344 (14)	0.00561 (12)	−0.00076 (14)
O1	0.0647 (9)	0.0366 (7)	0.0267 (6)	−0.0019 (6)	0.0068 (6)	0.0070 (5)
O2	0.0621 (8)	0.0302 (7)	0.0362 (7)	0.0129 (6)	0.0085 (6)	0.0010 (5)
O3	0.0469 (7)	0.0314 (6)	0.0462 (8)	−0.0108 (5)	0.0141 (6)	0.0010 (5)
O4	0.0577 (8)	0.0349 (7)	0.0264 (6)	−0.0101 (6)	0.0043 (5)	−0.0034 (5)
O5	0.0663 (9)	0.0270 (6)	0.0374 (7)	−0.0146 (6)	0.0043 (6)	−0.0016 (5)
O6	0.0745 (11)	0.0289 (7)	0.0416 (8)	−0.0115 (7)	0.0114 (7)	0.0064 (6)
C6	0.0364 (8)	0.0281 (8)	0.0275 (7)	0.0003 (6)	0.0078 (6)	0.0019 (6)
C7	0.0417 (8)	0.0323 (9)	0.0256 (8)	0.0013 (6)	0.0031 (6)	−0.0057 (6)
C8	0.0349 (7)	0.0246 (7)	0.0323 (8)	−0.0024 (6)	0.0027 (6)	−0.0067 (6)
C9	0.0303 (7)	0.0209 (6)	0.0250 (7)	−0.0012 (5)	0.0060 (5)	0.0000 (5)
C10	0.0310 (7)	0.0208 (6)	0.0257 (7)	−0.0020 (5)	0.0063 (5)	−0.0019 (5)
C11	0.0298 (7)	0.0220 (7)	0.0285 (8)	−0.0008 (5)	0.0083 (6)	0.0001 (5)
C12	0.0337 (7)	0.0245 (7)	0.0342 (8)	−0.0029 (6)	0.0084 (6)	0.0010 (6)
O1W	0.0539 (9)	0.0464 (9)	0.0617 (10)	−0.0046 (7)	0.0169 (8)	−0.0035 (8)

### Geometric parameters (Å, °)

**Table:**

N1—C1	1.342 (3)	S1—C9	1.7640 (16)
N1—C5	1.358 (3)	O1—C6	1.341 (2)
N1—H1N1	0.91 (3)	O1—H1O1	1.02 (3)
N2—C1	1.345 (3)	O5—C12	1.315 (2)
N2—H1N2	0.90 (3)	O5—H1O5	0.84 (4)
N2—H2N2	0.86 (3)	O6—C12	1.221 (2)
N3—C2	1.358 (3)	C6—C7	1.396 (3)
N3—H1N3	0.88 (3)	C6—C11	1.404 (2)
N3—H2N3	0.92 (3)	C7—C8	1.371 (3)
C1—C2	1.426 (3)	C7—H7	0.95 (3)
C2—C3	1.382 (3)	C8—C9	1.398 (2)
C3—C4	1.397 (4)	C8—H8	0.95 (3)
C3—H3	1.06 (3)	C9—C10	1.381 (2)
C4—C5	1.342 (4)	C10—C11	1.400 (2)
C4—H4	0.91 (4)	C10—H10	0.89 (2)
C5—H5	0.99 (4)	C11—C12	1.473 (2)
S1—O4	1.4484 (14)	O1W—H1W1	0.90 (3)
S1—O2	1.4542 (14)	O1W—H2W1	0.98 (5)
S1—O3	1.4631 (14)		
			
C1—N1—C5	124.4 (2)	O4—S1—C9	107.04 (8)
C1—N1—H1N1	118 (2)	O2—S1—C9	106.31 (8)
C5—N1—H1N1	117 (2)	O3—S1—C9	106.41 (8)
C1—N2—H1N2	115.9 (19)	C6—O1—H1O1	108.2 (16)
C1—N2—H2N2	118.6 (18)	C12—O5—H1O5	105 (2)
H1N2—N2—H2N2	120 (3)	O1—C6—C7	117.46 (16)
C2—N3—H1N3	124.8 (18)	O1—C6—C11	122.76 (16)
C2—N3—H2N3	120.1 (18)	C7—C6—C11	119.78 (15)
H1N3—N3—H2N3	113 (3)	C8—C7—C6	120.59 (15)
N1—C1—N2	119.15 (19)	C8—C7—H7	114.7 (16)
N1—C1—C2	118.33 (18)	C6—C7—H7	124.6 (16)
N2—C1—C2	122.50 (18)	C7—C8—C9	119.90 (15)
N3—C2—C3	123.45 (19)	C7—C8—H8	119.0 (17)
N3—C2—C1	119.73 (18)	C9—C8—H8	121.1 (17)
C3—C2—C1	116.80 (19)	C10—C9—C8	120.37 (15)
C2—C3—C4	121.9 (2)	C10—C9—S1	120.87 (12)
C2—C3—H3	116.2 (16)	C8—C9—S1	118.76 (12)
C4—C3—H3	121.7 (16)	C9—C10—C11	120.19 (14)
C5—C4—C3	119.5 (2)	C9—C10—H10	121.7 (15)
C5—C4—H4	120 (2)	C11—C10—H10	118.0 (15)
C3—C4—H4	120 (2)	C10—C11—C6	119.14 (14)
C4—C5—N1	118.9 (2)	C10—C11—C12	120.99 (15)
C4—C5—H5	127.2 (19)	C6—C11—C12	119.87 (14)
N1—C5—H5	113.4 (19)	O6—C12—O5	122.81 (16)
O4—S1—O2	112.20 (9)	O6—C12—C11	123.21 (16)
O4—S1—O3	112.80 (9)	O5—C12—C11	113.97 (15)
O2—S1—O3	111.58 (9)	H1W1—O1W—H2W1	124 (4)
			
C5—N1—C1—N2	−178.76 (19)	O2—S1—C9—C10	129.29 (13)
C5—N1—C1—C2	2.2 (3)	O3—S1—C9—C10	−111.65 (13)
N1—C1—C2—N3	175.05 (18)	O4—S1—C9—C8	−170.06 (13)
N2—C1—C2—N3	−3.9 (3)	O2—S1—C9—C8	−49.97 (14)
N1—C1—C2—C3	−3.3 (2)	O3—S1—C9—C8	69.09 (14)
N2—C1—C2—C3	177.74 (19)	C8—C9—C10—C11	0.1 (2)
N3—C2—C3—C4	−175.7 (2)	S1—C9—C10—C11	−179.10 (11)
C1—C2—C3—C4	2.5 (3)	C9—C10—C11—C6	1.7 (2)
C2—C3—C4—C5	−0.6 (4)	C9—C10—C11—C12	−179.31 (14)
C3—C4—C5—N1	−0.7 (4)	O1—C6—C11—C10	178.04 (16)
C1—N1—C5—C4	−0.2 (3)	C7—C6—C11—C10	−2.2 (2)
O1—C6—C7—C8	−179.37 (17)	O1—C6—C11—C12	−1.0 (2)
C11—C6—C7—C8	0.8 (3)	C7—C6—C11—C12	178.81 (15)
C6—C7—C8—C9	1.0 (3)	C10—C11—C12—O6	−174.36 (18)
C7—C8—C9—C10	−1.5 (2)	C6—C11—C12—O6	4.6 (3)
C7—C8—C9—S1	177.74 (14)	C10—C11—C12—O5	5.4 (2)
O4—S1—C9—C10	9.19 (15)	C6—C11—C12—O5	−175.63 (16)

### Hydrogen-bond geometry (Å, °)

**Table:**

*D*—H···*A*	*D*—H	H···*A*	*D*···*A*	*D*—H···*A*
N1—H1N1···O1W^i^	0.91 (3)	1.95 (3)	2.806 (3)	158 (3)
N2—H1N2···O1^i^	0.90 (3)	2.48 (3)	3.222 (2)	140 (2)
N2—H1N2···O1W^i^	0.90 (3)	2.28 (3)	3.060 (3)	145 (2)
N2—H2N2···O2^ii^	0.86 (3)	2.10 (3)	2.963 (2)	177 (3)
N3—H1N3···O2^ii^	0.89 (3)	2.10 (3)	2.970 (3)	168 (3)
N3—H2N3···O4^iii^	0.92 (3)	2.08 (3)	2.980 (3)	167 (3)
O1—H1O1···O6	1.03 (3)	1.75 (3)	2.625 (2)	141 (2)
O5—H1O5···O3^iv^	0.84 (3)	1.87 (3)	2.655 (2)	155 (3)
O1W—H1W1···O3^v^	0.89 (3)	1.94 (3)	2.757 (3)	151 (2)
O1W—H2W1···O4^iii^	0.98 (6)	1.89 (6)	2.838 (3)	162 (4)
C7—H7···O3^vi^	0.95 (3)	2.56 (3)	3.477 (2)	163 (2)

Symmetry codes: (i) *x*+1/2, −*y*+1/2, *z*−1/2; (ii) *x*+1/2, *y*−1/2, *z*; (iii) *x*+1/2, −*y*+1/2, *z*+1/2; (iv) *x*−1/2, *y*−1/2, *z*; (v) *x*−1/2, −*y*+1/2, *z*+1/2; (vi) *x*, −*y*+1, *z*+1/2.
